# Chemical leukoderma induced by dimethyl sulfate*

**DOI:** 10.1590/abd1806-4841.20164972

**Published:** 2016

**Authors:** Maya Valeska Gozali, Jia-an Zhang, Fei Yi, Bing-rong Zhou, Dan Luo

**Affiliations:** 1 Hospital of Nanjing Medical University, Nanjing – Jiangsu, China

**Keywords:** Chemical accidents, Chemical reactions, Skin diseases, Vitiligo

## Abstract

Chemical leukoderma occurs due to the toxic effect of a variety of chemical agents. Mechanisms include either destruction or inhibition of melanocytes. We report two male patients (36 and 51 years old) who presented with multiple hypopigmented macules and patches on the neck, wrist, and legs after exposure to dimethyl sulfate in a chemical industry. Physical examination revealed irregular depigmentation macules with sharp edges and clear hyperpigmentation around the lesions. History of repeated exposure to a chemical agent can help the clinical diagnosis of chemical leukoderma. This diagnosis is very important for prognosis and therapeutic management of the disease.

## INTRODUCTION

Chemical leukoderma refers to an acquired hypopigmented dermatosis induced by repeated exposure of the skin to specific chemical compounds.^1-5^ The majority of these chemicals are aromatic or aliphatic derivatives of phenols or catechols.^6,7^ We report two cases of chemical leukoderma induced by dimethyl sulfate. Dimethyl sulfate is an industrial alkylating agent used to convert chemical compounds – such as phenols, amines, and thiols – to the corresponding methyl derivatives.^8^ In addition to its carcinogenic properties, dimethyl sulphate is also known to be very irritant to mucous membranes because of its rapid hydrolysis in water to methanol and sulfuric acid. The primary routes of potential occupational uptake for this substance are inhalation and dermal contact.^8,9^

## CASE REPORT

Two male workers aged 36 and 51 years presented with multiple hypopigmented macules and patches on the neck, wrist, and legs. They had no personal or family history of vitiligo or any other autoimmune disease. They had worked in a chemical industry for approximately one year. Six months before presenting to treatment at the local hospital, their face, wrist, and other unprotected body parts were exposed to vapor coming from a dimethyl sulfate leakage. They noticed a consequent burning sensation and swelling on the face, swelling and pain on the wrist and ulcerated skin lesions. The treated skin presented contractures, hyperpigmentation and scarring. The skin lesions crusted then shed off. After that, multiple depigmentation spots appeared on the exposed area.

**Figure 1:**
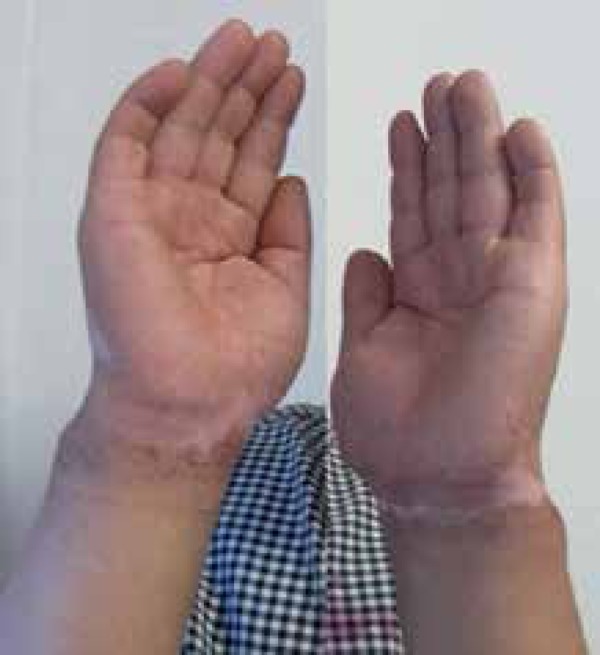
36-year-old man presented with visible irregular depigmentation macules with sharp edges and clearly visible hyperpigmentation around the lesions on the wrist

**Figure 2:**
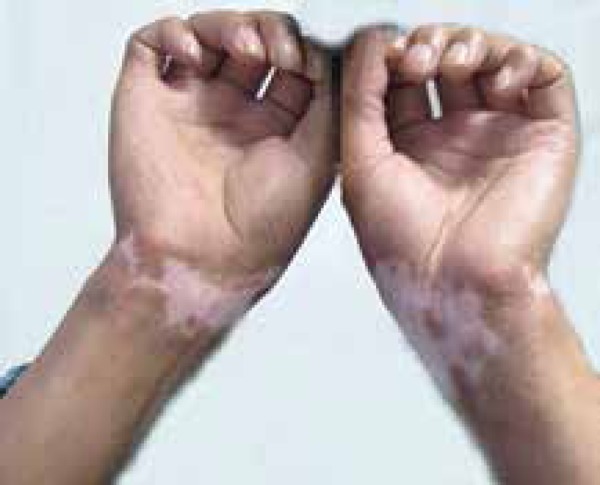
51-year-old man presented with visible irregular depigmentation macules with sharp edges and clearly visible hyperpigmentation around the lesions on the wrist

**Figure 3:**
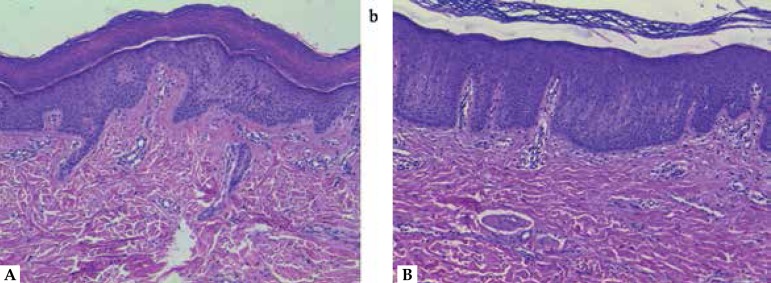
Histopathology examination showed stratum corneum slightly thicker than normal skin and partial or complete melanin loss due to the absence of melanocytes in both patients (a and b) (Haematoxylin and eosin staining, magnification ×20)

Physical examination revealed irregular depigmentation macules with sharp edges and clear hyperpigmentation around the lesions on the wrists of both patients (Figures 1 and 2). We observed no abnormalities besides the skin lesions on general physical examination and diagnosed chemical leukoderma. Histopathology examination showed stratum corneum slightly thicker than normal skin and partial or complete melanin loss due to the absence of melanocytes (Figure 3).

## DISCUSSION

Chemical leukoderma is also designated as contact leukoderma or occupational leukoderma.^4^ Although all age groups (from pediatric to geriatric, including neonates) may be affected by chemical leukoderma, adults have a much higher incidence of the disease.^1,4^ The first case of toxic leukoderma following occupational contact was reported in 1939 in workers exposed to monobenzylether of hydroquinone present in rubber gloves. Since then, a variety of chemicals causing chemical leukoderma have been reported.^2,5^ The majority of these chemicals are aromatic or aliphatic derivatives of phenols and catechols. Dimethyl sulfate (CH3)2SO4 is a methylating agent used industrially in the synthesis of pharmaceuticals, perfumes and pesticides to convert compounds such as phenols, amines and thiols.^9^ Phenolic/catecholic derivatives induce melanocyte toxicity via tyrosinase-related protein-1, which catalytically converts these chemicals within melanocytes and leads to the production of reactive oxygen species. In normal melanocytes, this oxidative stress has been shown to trigger free radical scavenging in order to prevent apoptosis of the melanocyte.^4,5^ Boissy *et al.* hypothesized that the genetic inability of melanocytes to respond to tyrosinase-related protein-1 oxidative stress may underlie the etiology of chemical leukoderma.^6^ This genetic susceptibility explains why only a certain subset of patients will develop chemical leukoderma upon exposure to a given compound.

The areas of involvement depend upon the route of exposure. Lesions are frequently widespread, including areas of direct skin contact and accidentally transferred from hand to other parts of the body.^2^ Face and scalp were the most common and least affected sites, respectively, in chemical leukoderma. On the face, the eyelids were a major area of involvement. This probably originates from greater penetration of the offending toxic chemicals through the thinner skin of the face (eyelids are the thinnest and the scalp is the thickest). However, the hands and feet, although composed of much thicker skin, showed a high incidence of chemical leukoderma, probably due to a higher rate of exposure.^4^ Although chemical leukoderma usually is not associated with systemic disease, concomitant cases of thyroid disease, hepatosplenomegaly, and transaminitis have been reported.^5^

Chemical leukoderma should be considered in the differential diagnosis of every case of idiopathic vitiligo or leukomelanoderma. Chemical leukoderma develops not only at the site of chemical contact, but also remotely. The mechanism responsible for this distant spread of the disease could be sensitization, autotransfer, or heterotransfer of the chemical from patients themselves and people close to them.^4^ Chemical leukoderma can be diagnosed clinically by a history of repeated exposure to a known or suspected depigmenting agent at the primary site, distribution of macules corresponding to chemical exposure, and the presence of numerous acquired peasized macules.^4^ There are no confirmatory tests for chemical leukoderma.^2^ Our patients presented visible irregular depigmentation macules with sharp edges and clearly visible hyperpigmentation around the lesions. Skin lesions were first developed in occupationally exposed sites. Histopathology examination revealed vitiligo-like hypomelanosis. Patients had no personal or family history of vitiligo or autoimmune disease. As a result, chemical leukoderma was diagnosed.

Chemical leukoderma is histologically identical to vitiligo and may be hard to distinguish clinically except by specific area exposure history-.^3-5,10^ This diagnosis is very important to assess the prognosis and manage therapeutically as chemical leukoderma shows a better outcome than vitiligo.^1^ The most important principle of treatment is discontinuation of the irritant. Avoidance of the causative agent may lead to spontaneous repigmentation, but treatments for chemical leukoderma parallel those of vitiligo. These include psoralen and long-wave ultraviolet radiation or UVB phototherapy, epidermal surgical grafting, and topical or intralesional corticosteroids. ^2,3^
